# Infrared and visible image fusion method of dual NSCT and PCNN

**DOI:** 10.1371/journal.pone.0239535

**Published:** 2020-09-18

**Authors:** Chunming Wu, Long Chen

**Affiliations:** 1 Key Laboratory of Modern Power System Simulation and Control & Renewable Energy Technology, Ministry of Education (Northeast Electric Power University), Jilin, China; 2 Department of Electrical Engineering, Northeast Electric Power University, Jilin, China; University of Engineering & Technology, Taxila, PAKISTAN

## Abstract

To solve the problem that the details of fusion images are not retained well and the information of feature targets is incomplete, we proposed a new fusion method of infrared (IR) and visible (VI) image—IR and VI image fusion method of dual non-subsampled contourlet transform (NSCT) and pulse-coupled neural network (PCNN). The method makes full use of the flexible multi-resolution and multi-directional of NSCT, and the global coupling and pulse synchronization excitation characteristics of PCNN, effectively combining the features of IR image with the texture details of VI image. Experimental results show that the algorithm can combine IR and VI image features well. At the same time, the obtained fusion image can better display the texture information of image. The fusion performance in contrast, detail information and other aspects is better than the classical fusion algorithm, which has better visual effect and evaluation index.

## 1 Introduction

The status of image fusion technology is irreplaceable in the historical process of image technology. So far, image fusion has penetrated into various fields such as computer vision [1], medical image [2–4], and electricity [5]. Image fusion is mainly to combine the information of two or more related multi-source images into a single image through an appropriate algorithm. Generally, there are two types of fusion source images: images from the same sensor and images from different sensors. Images from different sensors are called heterogeneous images. Each type of sensor has its own unique attributes, and the different attributes of the two sensors will lead to great differences in the images obtained, but these images have complementary characteristics. Therefore, the fusion of heterogeneous images has more research value. There are many kinds of fusion of heterogeneous images, but among them, the VI and IR image are the most widely studied and applied. The technology effectively combines the advantages of the two kinds of images, making the fused image with higher contrast and stronger background. This technology has something in common with the precise positioning of target detection, so it provides great convenience for target detection.

There have been a lot of researches on the fusion of VI and IR image, but the existing researches mainly focus on how to improve the infrared characteristics and how to remove the artifacts caused by spectral differences, and fail to consider the fine texture in visible image. In practical engineering applications, background details are sometimes as important as infrared features. Therefore, it is not comprehensive to consider only the good acquisition of infrared targets without considering the background details. At present, there are two trends in image fusion: one is the classical fusion based on pixel features, and the other is the use of artificial neural network to fuse images. In the classical pixel-based fusion algorithm, the discrete wavelet transform (DWT) bears the brunt. After the unremitting efforts of the later researchers, NSCT [6] and NSST [7, 8] also arised at the historic moment. Bhatnagar et al. [9] studied medical images and applied the indicated contrast in image fusion. In [10], fu et al. used the sparse matrix to guide the fusion rules of high and low frequency subband coefficients, and highlighted the infrared characteristics of the fusion results. Due to the differences in images of different imaging mechanisms and the artifact problem of fusion images, Cheng et al. [11] introduced singular value decomposition into image fusion to solve this problem. Li et al. [12] improved the PCNN by stimulating the PCNN with spatial frequency and adjusting the parameters with average gradient to improve the image quality. With the development of machine learning and artificial intelligence, many scholars have applied machine learning and neural network to image fusion. Nowadays, machine learning and artificial intelligence are developing rapidly. For example, Shukla et al. [13] proposed a new model integrating the advantages of convolutional neural network, recursive neural network and mixed density network, and realized the prediction of drug interaction score. Pathak et al. [14] established a deep metastatic learning classification model to classify COVID-19 diseases. Therefore, many scholars apply machine learning and convolutional neural network to image fusion. Kaur et al. [15] use a deep belief network to evaluate the data set through various feature extraction techniques, and then use feature selection techniques to select potential features to achieve medical image fusion. Kong et al. [16] combined the advantages of deep convolutional neural network and extreme deep learning to establish a fusion model and complete the fusion process of multi-focus images. Space and time fusion is an important means to provide intensive time series for earth observation with high spatial resolution, Liu et al. [17] achieved fast and accurate space and time image fusion by using extreme machine learning. The combination of image fusion and machine learning can also recognize biometric characteristics. Li et al. [18] proposed a virtual reality gesture recognition algorithm based on image information fusion and achieved a recognition success rate of 96.17%. The above researchers have made great contributions to the development of image fusion technology, but they have not considered the background details of the fusion image. Such as: the method [10] only highlights the infrared features, and the method in [11] solves the problem of artifact. It also does not consider the texture details in the VI image. Although the method in [12] makes improvements on the basis of target information and detail information, it still emphasizes feature targets rather than detail information.

In VI images, due to the unique features of PCNN, global coupling and pulse synchronization, this feature can effectively help extract background information. Therefore, we propose an IR and VI image fusion method based on dual NSCT and PCNN, which not only improves the acquisition of texture details, but also retains infrared features, improving the resolution and fusion quality of the fusion image. The method needs to fuse the image twice. In the first fusion, the high and low frequency coefficients are obtained by the large modulus rule and the adaptive fuzzy logic rule respectively. The PCNN fusion rule is applied in the secondary fusion. Firstly, the low-frequency sub-band image is filtered. The external excitation of PCNN is triggered by the processed coefficient feature and direction information feature weighting in the low-frequency fusion, and directly triggered by the pixel gray value in the high-frequency coefficient fusion.

Experiments show that this method can fuse IR and VI images well. At the same time, we compare performance with traditional methods in the form of quantitative indicators. Our contributions in this paper are as follows:

1. We analyze the problems existing in the existing algorithms and propose a new IR and VI image fusion algorithm.
2. The proposed algorithm can fully extract the background information in VI image and improve the quality of the fusion image.
3. We propose an image feature weighting mechanism based on Mean spectral radius, and apply orientation information and image filtering to image fusion in a weighted way.
4. We compare the traditional method with the proposed image fusion method from quantitative and qualitative perspectives.

The paper is organized as follows. In section 2, the principles of NSCT and PCNN are briefly summarized. Section 3 describes our proposed algorithm in detail. Section 4 gives the experimental results and compares them with several classical algorithms such as DWT, PCNN and NSCT_PCNN. Finally, the conclusion is given.

## 2 Preliminaries

NSCT is the most common method of image decomposition, and translation invariance is the most valuable feature of NSCT. PCNN is a network model mechanism based on the bionic vision cortex in the cat brain. Therefore, NSCT and PCNN are commonly used in image processing.

### 2.1 NSCT

**Fig 1:**
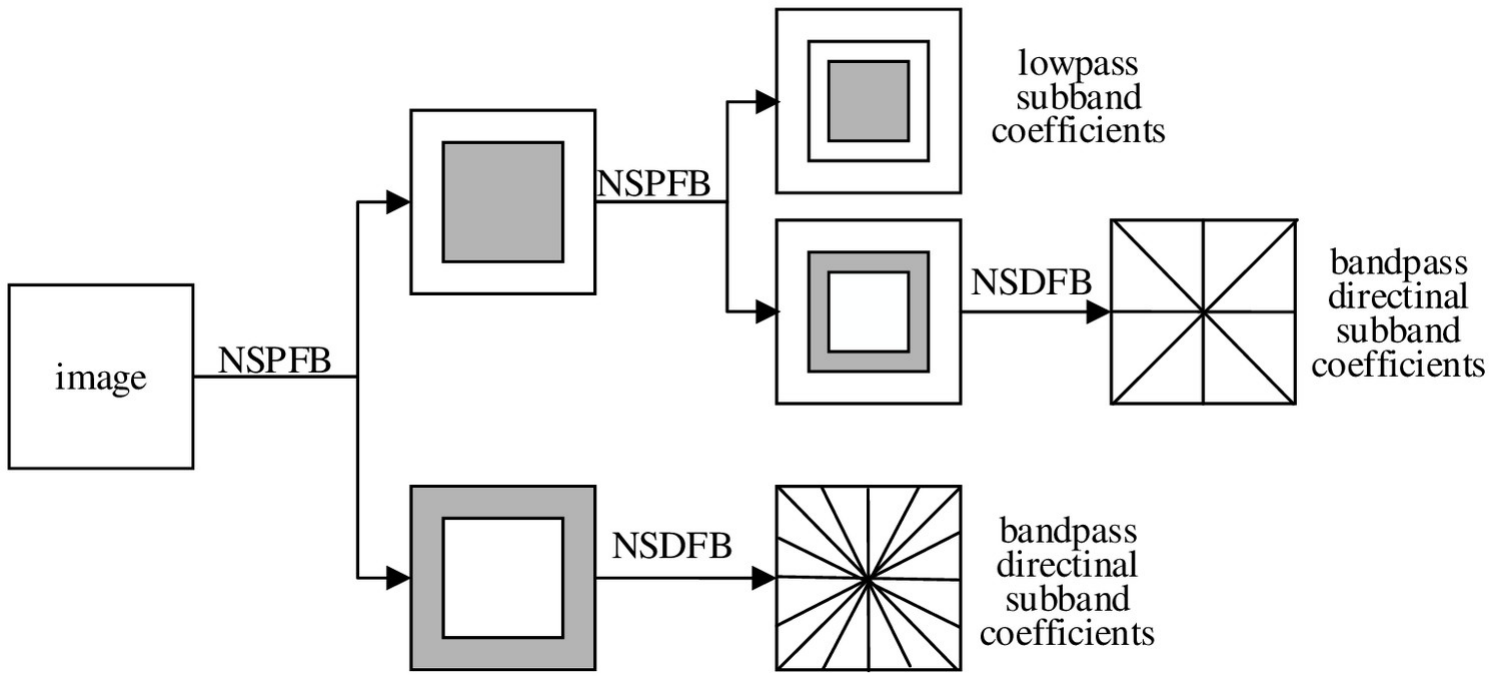
Two-level decomposition of NSCT.

**Fig 2:**
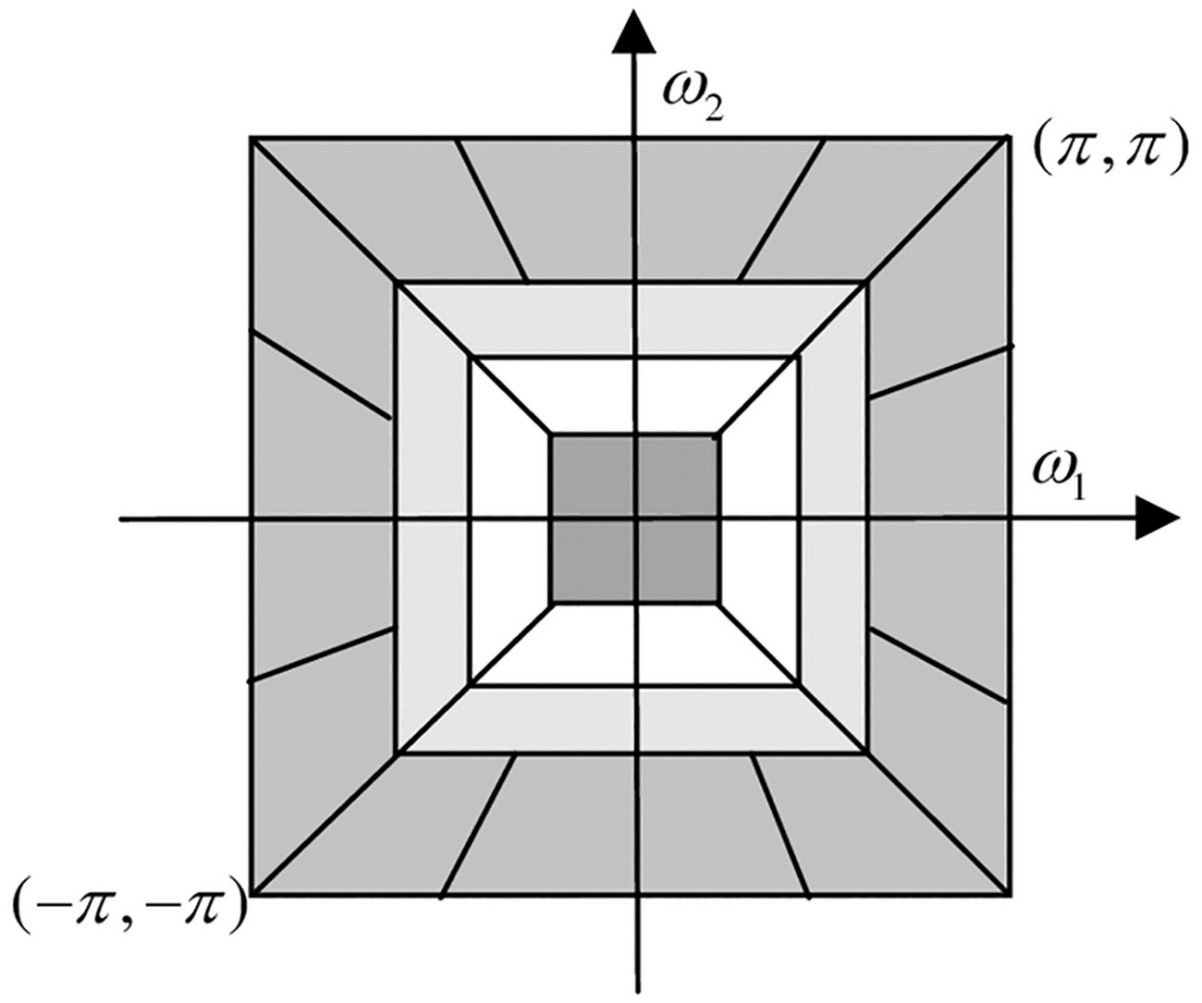
Schematic diagram of two-dimensional frequency division.

The contourlet transform is improved by the researchers to obtain NSCT, which is composed of two parts: one is non-subsampling pyramid filter bank (NSPFB) and another is non-subsampling direction filter bank (NSDFB) [19]. Generally, there are two operations of up-sampling and down-sampling in multi-scale decomposition, but this operation is removed in NSPFB. Moreover, NSDFB synthesizes the coefficients of NSCT from the singularities distributed in the same direction, thus reducing the distortion during sampling. NSCT consists of two steps: first, NSPFB decomposes the image acquired by the sensor, and then NSDFB decomposes the band-pass image of various scales generated in the previous step. After the processing of the above two steps, the images of different scales and directions are obtained respectively. The two-level decomposition of NSCT is shown as Fig 1; the schematic diagram of two-dimensional frequency domain division is described as Fig 2.

### 2.2 PCNN

**Fig 3:**
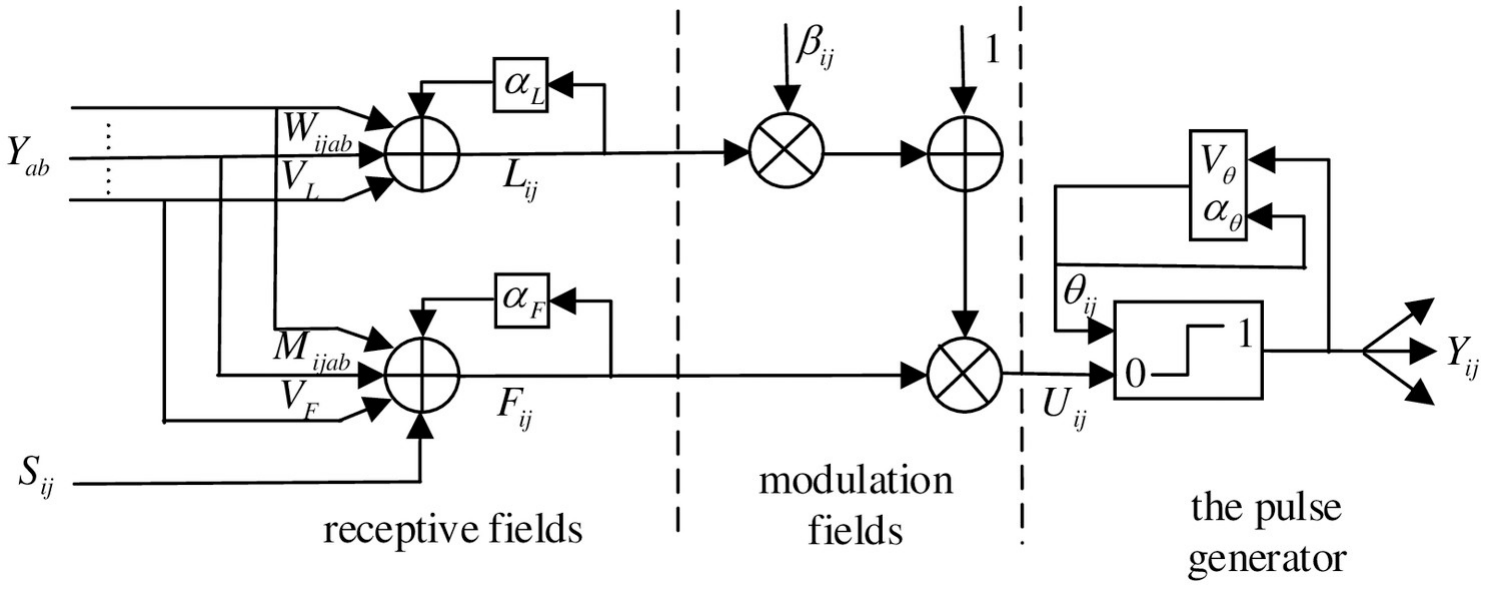
Basic structure of pulse-coupled neuron.

PCNN is a network that generates signals in the form of triggers based on the physiological visual nervous system and has the function of signal feedback. It has no learning process or training process, and can extract useful information effectively for complex environments. Receptive fields, modulation fields, and pulse generators form most basic components of a single neuron [20]. The basic structure of pulse-coupled neurons is shown as Fig 3 and the corresponding expression is described as:

$$F_{ij}[\text{n}]=\text{exp}(-\alpha_{F})F_{ij}[\text{n}-1]+{\text{V}}_{F}\sum_{kl}M_{ijkl}Y_{kl}[\text{n}-1]+S_{ij}$$ (1)

$$L_{ij}[\text{n}]=\text{exp}(-\alpha_{L}){\text{L}}_{ij}[\text{n}-1]+V_{L}\sum_{kl}W_{ijkl}Y_{ij}[\text{n}-1]$$ (2)

$$U_{ij}[\text{n}]=F_{ij}[\text{n}](1+\beta L_{ij}[\text{n}])$$ (3)

$$\theta_{ij}[\text{n}]=\text{exp}(-\alpha_{\theta })\theta_{ij}[\text{n}-1]+V_{\theta }Y_{ij}[\text{n}]$$ (4)

$$Y_{ij}[\text{n}]=\left\{ \begin{array}{l} 1,U_{ij}[\text{n}]\geq \theta_{ij}[\text{n}]\\ 0,U_{ij}[\text{n}]<\theta_{ij}[\text{n}] \end{array}\right.$$ (5)

where *S*_*ij*_ is the excitation produced by the surrounding neurons; *F*_*ij*_ is feedback signal; *L*_*ij*_ is the link input; *U*_*ij*_ is the convolution process of the signal inside the neuron; *θ*_*ij*_ is a threshold value that varies according to the actual situation; *Y*_*ij*_ represents the excitation input corresponding to the image pixel point; n is iterative times; *M*_*ijkl*_ and *W*_*ijkl*_ are the connection matrix between feedback and coupling links domains in receptive fields respectively; *V*_*F*_ and *V*_*L*_ are amplification factors; *V*_*θ*_ is the magnification factor of dynamic threshold *θ*; *α*_*F*_, *α*_*L*_ and *α*_*θ*_ are decay time constants; *β* is the linking strength.

## 3. The proposed fusion algorithm

**Fig 4:**
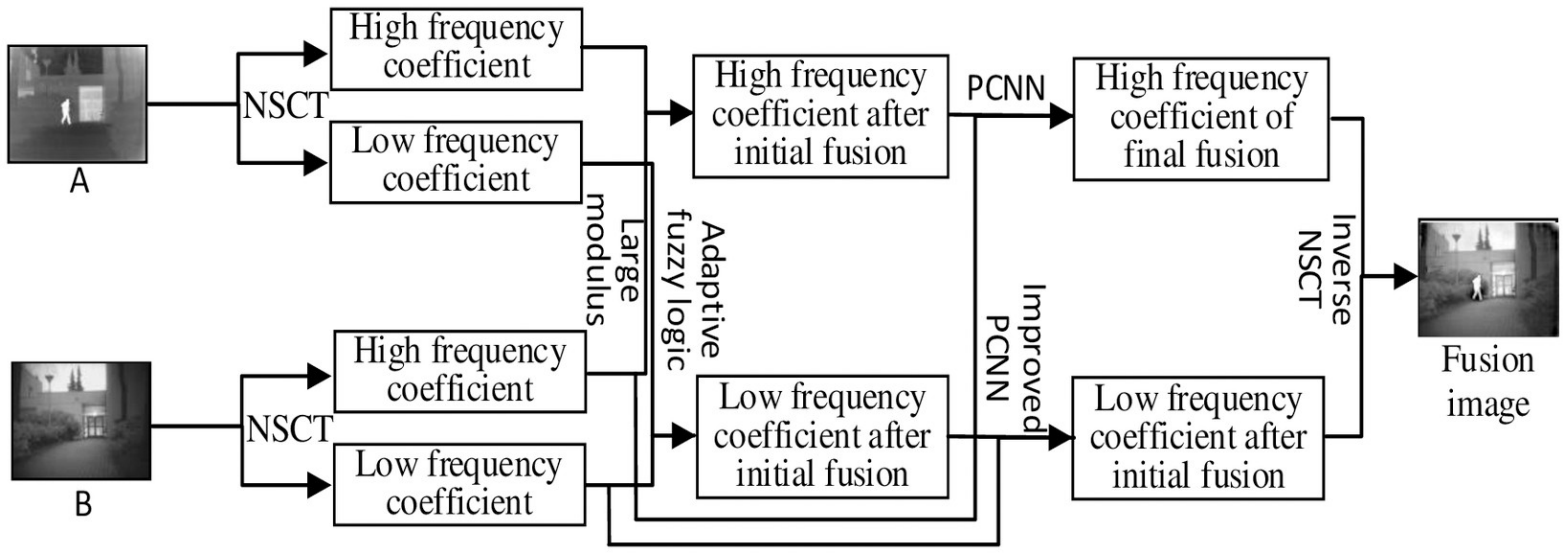
Fusion process frame.

The detailed implementation process of the proposed algorithm, fusion rules and related basic theories applied in the fusion rules, including orientation information, image filtering and average spectral radius, are introduced in this section. The fusion process framework is shown as Fig 4.

### 3.1 Low-frequency fusion rules of initial fusion

The energy of the image is not uniformly distributed. The low-frequency part gathers most of the energy because it carries contour and feature information. Therefore, the adaptive fuzzy logic algorithm is adopted for the low frequency sub-band. Fuzzy logic is a method of describing fuzzy concepts with precise numerical language. Those fuzzy concepts that cannot be quantitatively described can be expressed by fuzzy sets, and different fuzzy sets are represented by constructing different membership functions. The fuzzy membership functions are defined as follows:

$$C=\{x,\mu_{C}(x)|x\in U\}$$ (6)

where *U* is universe (a collection of objects); *x* is an element in the domain; *C* is the fuzzy set in *U*; *μ*_*C*_(*x*) is the membership of *C*, which value is between 0 and 1.

In this paper, an adaptive weighted average fusion rule based on fuzzy logic is proposed and gaussian membership function is introduced from the adaptive weighting coefficient. The expression is described as follows:

$$\eta_{0}(i,j)=\text{exp}[-\frac{{\text{(}D\text{(}i,j\text{)}-\mu \text{)}}^{2}}{k{\left(2\sigma \right)}^{2}}]$$ (7)

$$\eta_{1}(i,j)=1-\eta_{0}(i,j)$$ (8)

where *D*(*i*, *j*) is the low-frequency sub-images coefficient; *μ*, *σ* are the mean and variance of A’s low-frequency sub-image pixels, respectively. *k* is the adjustment parameter, and usually takes its empirical value, which is 1.5. Its low frequency fusion rule can be described as

$$D_{F}(i,j)=\eta_{0}(i,j)D_{A}(i,j)+\eta_{1}(i,j)D_{B}(i,j)$$ (9)

where *D*_*A*_(*i*, *j*) and *D*_*B*_(*i*, *j*) are the low-frequency sub-band coefficient of IR and VI images respectively, *D*_*F*_(*i*, *j*) represents the low-frequency sub-images coefficient after fusion.

### 3.2 High-frequency fusion rules of initial fusion

The texture and edge information of the image is usually carried by the high-frequency part of the image. Each coefficient after decomposition is a quantitative description of the image details, so the coefficient with a large absolute value is an approximate representation of the source image details. Therefore, as long as the coefficients with large absolute values are preserved, then the features of the source image are preserved. We adopt the large modulus algorithm based on the feature points. The expression is described as follows:

$$E_{F}^{H}\text{(}i,j\text{)}=\left\{ \begin{array}{l} E_{A}^{H}\text{(}i,j\text{)},\text{if}|E_{A}^{H}\text{(}i,j\text{)}|\geq |E_{B}^{H}\text{(}i,j\text{)}|\\ E_{B}^{H}\text{(}i,j\text{)},\text{if}|E_{A}^{H}\text{(}i,j\text{)}|<|E_{B}^{H}\text{(}i,j\text{)}| \end{array}\right.$$ (10)

where $E_{F}^{H}(i,j)$ is high-frequency sub-images coefficient after fusion; $E_{A}^{H}(i,j)$ and $E_{B}^{H}(i,j)$ are the coefficients of A and B at position (*i*, *j*), respectively.

### 3.3 Orientation information

The description of image texture information and edge features can be completed by directional information (OI), which can present the segmented smoothing characteristics of the image. Assuming that *I*(*i*, *j*) is the pixel value at (*i*, *j*), the orientation information of the window size (2*w*_*x*_ + 1) × (2*w*_*y*_ + 1) centered on (*i*, *j*) is calculated as follows:

$$OI(i,j)=\underset{0^{{}^{\circ}}\leq \theta \leq 180^{{}^{\circ}}}{\text{max}}(d_{\theta })-\underset{0^{{}^{\circ}}\leq \theta \leq 180^{{}^{\circ}}}{\text{min}}(d_{\theta })$$ (11)

among them

$$d_{\theta }=|\sum_{(\text{i},\text{j})\in \text{AL}}I(i,j)-\sum_{(\text{i},\text{j})\in \text{AR}}I(i,j)|$$ (12)

**Fig 5:**
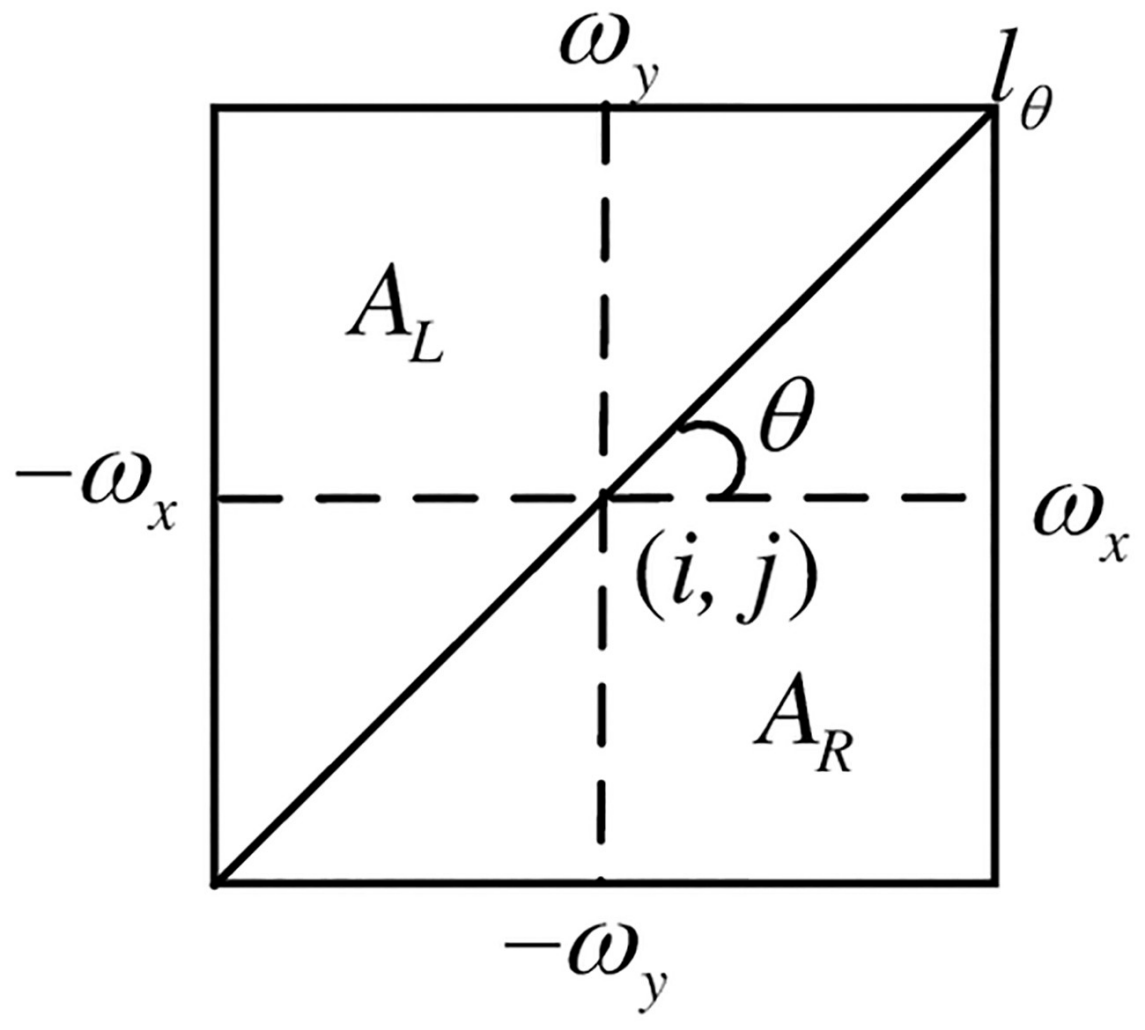
Schematic calculation of direction information.

*AL* and *AR* represent the left and right areas of the window. The calculation of the direction information is shown as Fig 5.

### 3.4 Image filtering

Image filtering can not only preserve the details of the image, but also inhibit the noise of the target image. The results of image filtering have a direct influence on the validity and reliability of image processing and analysis. Mean filtering can effectively filter the additive noise in the image, but it can’t locate the image features well. Edge filtering is used to find a set of pixel points in the image with sharp brightness variation, which is often represented by the contour and can accurately locate the position of image feature points. Therefore, the low-frequency sub-band are processed by mean filtering and edge filtering respectively, and the optimal feature points after filtering are selected as the feature points of the low frequency sub-band, which can be calculated by

$$M_{s}(i,j)=\left\{ \begin{array}{l} M_{2}(i,j),\text{if}|M_{1}(i,j)|\geq |M_{2}(i,j)|\\ M_{2}(i,j),\text{if}|M_{1}(i,j)|<|M_{2}(i,j)| \end{array}\right.$$ (13)

where *M*_*s*_(*i*, *j*) is the selected feature point; *M*_1_(*i*, *j*) and *M*_2_(*i*, *j*) are the feature points after filtering. The convolution mask of mean filtering and edge detection is described as follows:

$$\left[\begin{array}{ccc} 0.11 & 0.11 & 0.11\\ 0.11 & 0.12 & 0.11\\ 0.11 & 0.11 & 0.11 \end{array}]\;[\begin{array}{ccc} -1 & -1 & -1\\ -1 & -9 & -1\\ -1 & -1 & -1 \end{array}\right]$$

### 3.5 Mean spectral radius

The overall statistical situation of matrix elements is difficult to present perfectly with a single eigenvalue. The mean spectral radius (MSR) [21] uses all the eigenvalue data of the random matrix to reflect its own overall statistics. MSR is a linear eigenvalue statistic of the random matrix, which can also be expressed by the average distance from all the eigenvalues of the random matrix to the origin of the complex plane. It can be obtained by

$$k_{\text{MSR}}=\frac{1}{\text{n}}\sum_{i=1}^{\text{n}}\left|\lambda_{i}\right|$$ (14)

where *λ*_*i*_(*i* = 1, ⋯, *n*) is the eigenvalue of the random matrix *X*, and |*λ*_*i*_| is the modulus corresponding to the eigenvalue, besides, n is the number of eigenvalues.

### 3.6 Fusion steps

The specific steps of the fusion algorithm are as follows:

Step1. IR image A and VI image B were decomposed by NSCT, obtained the low-frequency sub-images coefficients *L*_*A*_ and *L*_*B*_, high-frequency sub-images coefficient $H_{A_{i}}^{k}$ and $H_{B_{i}}^{k}$, where direction number is *k* = 1, 2, ⋯, 2^*i*^, series is *i* = 1, 2, ⋯.
Step2. Applying adaptive fuzzy logic fusion rules for low-frequency sub-images coefficient, applying large fusion rule with module values for high-frequency sub-images coefficient. After initial fusion, high and low frequency sub-image coefficients $H_{F1_{i}}^{k}$ and *L*_*F*1_ were obtained respectively.
Step3. So as to effectively supplement the missing information in the initial fusion image, the low-frequency sub-image coefficients of the initial fusion image and B were processed according to the rules of image filtering and direction information. After processing the low frequency sub-images coefficient obtained $L_{\text{F}1}^{\text{IF}}$, $L_{\text{F}1}^{\text{OI}}$ and $L_{\text{B}}^{\text{IF}}$, $L_{\text{B}}^{\text{OI}}$. Based on the rule of mean spectral radius, the processed low-frequency sub-images coefficient is weighted as the external excitation of PCNN neurons. Its expression can be calculated by
  $$L=\frac{k_{\text{MSR}}^{\text{IF}}}{k_{\text{MSR}}^{\text{IF}}+k_{\text{MSR}}^{\text{OI}}}*L_{*}^{\text{IF}}+\frac{k_{\text{MSR}}^{\text{OI}}}{k_{\text{MSR}}^{\text{IF}}+k_{\text{MSR}}^{\text{OI}}}*L_{*}^{\text{OI}}$$ (15)
  where *L* is the weighted low-frequency sub-images coefficient; $k_{\text{MSR}}^{\text{IF}}$ and $k_{\text{MSR}}^{\text{OI}}$ are the average spectral radius of low-frequency sub-images coefficient after image filtering and direction information rule processing, respectively. * in $L_{*}^{\text{IF}}$ and $L_{*}^{\text{OI}}$ represents the initial fusion image F1 or visible image B.
Step4. In order to fully reflect the correlation between the source images, PCNN is stimulated by the pixel gray value of the source image to process the high-frequency sub-images.
Step5. After the above steps, the processed high and low frequency sub-band images are obtained. Finally, do inverse NSCT to get the final fusion result.

## 4. Experimental results and analysis

**Fig 6:**
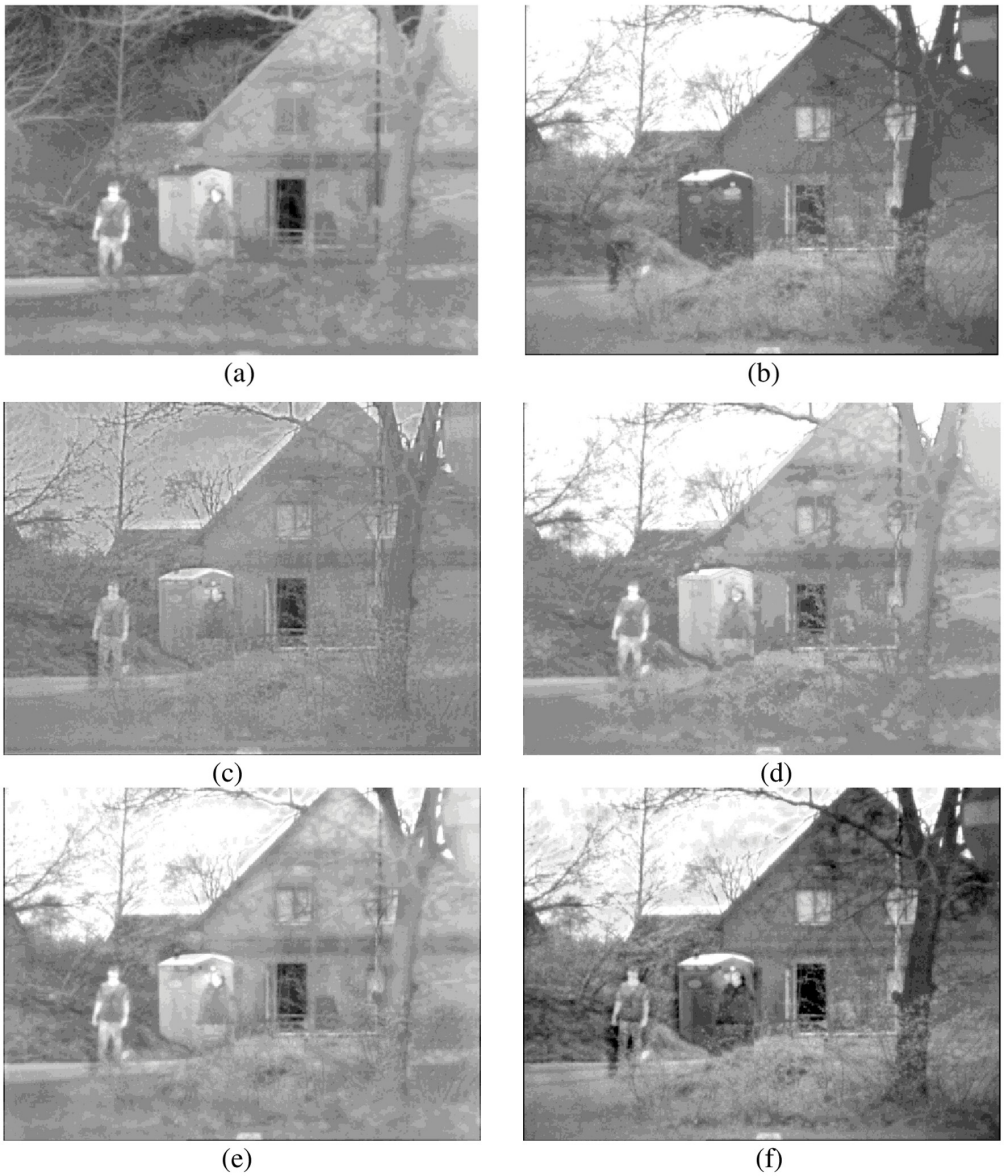
The first group of experimental results. (a) IR image, (b) VI image, (c) DWT, (d) PCNN, (e) NSCT_PCNN, (f) Proposed method.

**Fig 7:**
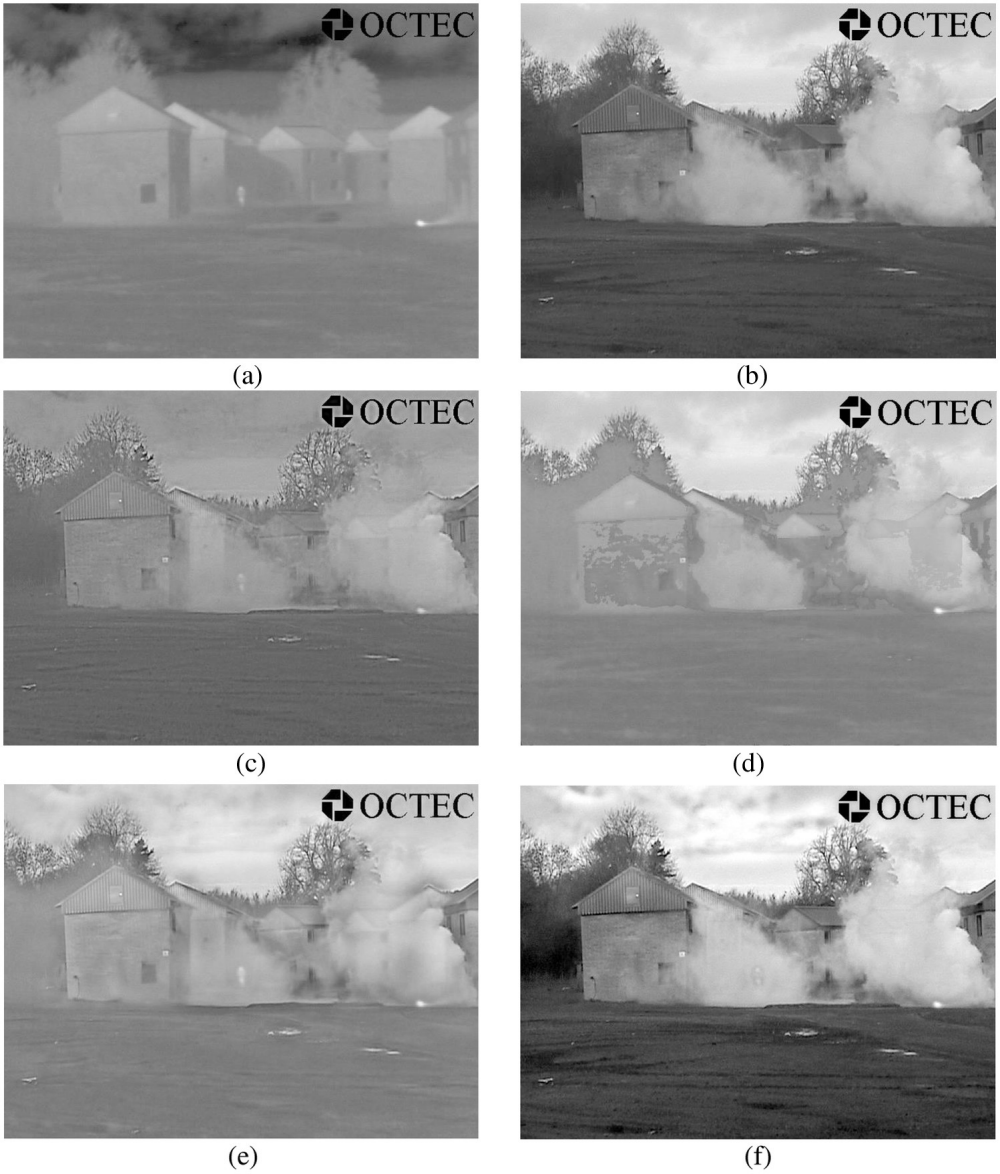
The second group of experimental results. (a) IR image, (b) VI image, (c) DWT, (d) PCNN, (e) NSCT_PCNN, (f) Proposed method.

**Fig 8:**
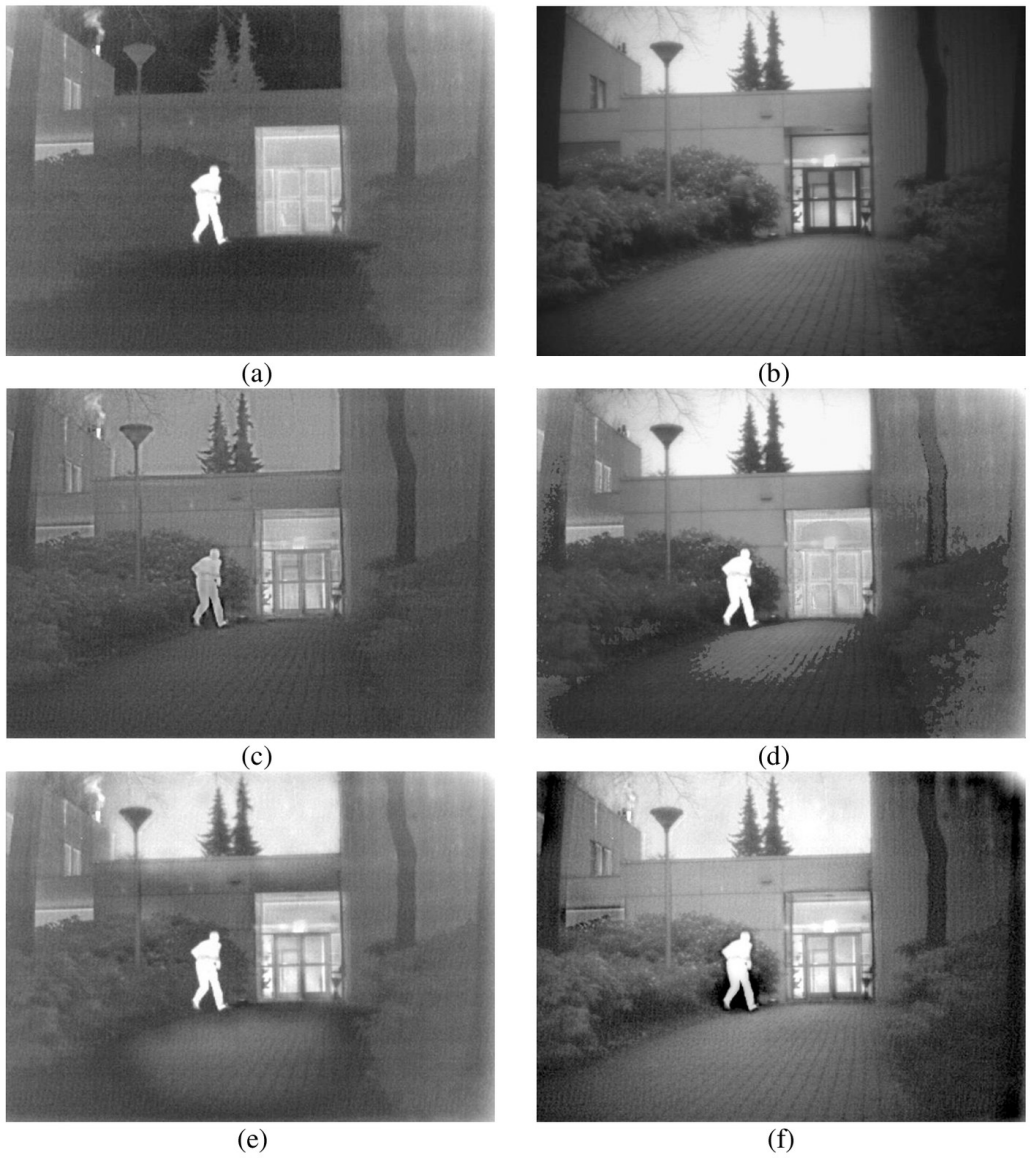
The third group of experimental results. (a) IR image, (b) VI image, (c) DWT, (d) PCNN, (e) NSCT_PCNN, (f) Proposed method.

The rationality of each proposed algorithm has to be verified. For this reason, all experiments in this paper were conducted under the Windows 10 operating system, with the CPU frequency of 2.20 GHz and memory of 8.00 Gb. Simulation was conducted on the Matlab R2018b platform. In this paper, the "maxflat" filter and "dmaxflat7" filter were used for the NSCT at the initial fusion, and the "9/7" filter and "pkva" filter were used for the NSCT at the second fusion. Adaptive PCNN model parameter settings: n = 200, *α*_*L*_ = 0.06931, *α*_*θ*_ = 0.2, *V*_*L*_ = 1.0, *V*_*θ*_ = 20. In order to better verify our method, we compared it with DWT [22], PCNN [23], and NSCT_PCNN [24]. DWT adopts three-layer "db2" wavelet; *β* = 0.2 in PCNN and NSCT_PCNN methods. A good algorithm should have breadth as well as depth, so we selected three images of different environments. The experimental images are from open access data sets, can be obtained through the website: https://figshare.com/articles/TNO_Image_Fusion_Dataset/1008029. The three groups of pictures show people in the porch, people walking into the building, and smoke-covered factories. The original size of the three groups of experimental pictures is 768×576, 750×562 and 620×450. The fusion results of three methods based on DWT, PCNN and NSCT_PCNN are shown in Figs 6 to 8.

As can be seen from Figs 6 to 8, DWT results are not ideal either in terms of contrast or overall effect, and they fail to show good goals and background. Although the results of NSCT_PCNN showed the feature target well, the texture details, such as shrubs and houses, were not shown well. The results of PCNN method did not achieve good results in either feature target or texture details. The edge contour of the fusion images obtained by our method are clear, the images texture is basically consistent with the source images, and the character features are the most significant. Compared with other fusion algorithms, our proposed method achieves better results in both visual effects and texture details.

In most cases, human vision is indistinguishable from small differences, so it is not easy to appraise the fusion results in terms of visual perception. Therefore, generally speaking, the results of image fusion should not only be evaluated subjectively, but also analyzed objectively. This article selects four objective quality indexes as evaluation standard: (1) information entropy (IE) [25, 26], (2) standard deviation (SD) [27], (3) average gradient (AG) [28], and (4) space frequency (SF) [29]. The quantitative analysis of the experimental results is shown in Tables 1–3. The bold black font represents the best results of several algorithms.

**Table 1:** Objective evaluation of the first group of images.

	IE	SD	AG	SF
DWT	6.5787	25.6475	5.4595	12.0034
PCNN	6.5950	47.1038	4.3125	10.4638
NSCT_PCNN	7.0123	47.6294	4.6988	11.4942
Proposed method	**7.2322**	**60.3081**	**6.5783**	**13.9361**

**Table 2:** Objective evaluation of the second group of images.

	IE	SD	AG	SF
DWT	6.6670	32.5021	4.8464	8.9062
PCNN	7.1859	61.6588	4.5460	9.9035
NSCT_PCNN	7.1598	52.1811	4.6812	8.8897
Proposed method	**7.2982**	**64.7182**	**6.1197**	**11.1466**

**Table 3:** Objective evaluation of the third group of images.

	IE	SD	AG	SF
DWT	6.4773	30.5938	6.2007	15.7788
PCNN	5.5219	38.7337	5.3596	16.3384
NSCT_PCNN	6.8221	38.8706	5.9005	16.1174
Proposed method	**7.1368**	**73.9785**	**8.7715**	**20.6919**

In Table 1, we can see that the values of SD, IE, AG and SD are higher than other three methods. The higher SD of our algorithm indicates that the distribution of gray value is uniform, which can achieve better detail conversion and clarity. At the same time, both AG and SF are higher than other algorithms, indicating that our algorithm can better express the detail transformation and hierarchical transformation. The IE is higher than other algorithms, which shows that the fusion result is closer to the source image. In the second and third experiments, satisfactory results were obtained for all four quantitative indicators. It fully shows that the method we proposed keeps the edge contour high, the image details are richer, and the overall effect is better, which is consistent with the human visual perception. In addition, in terms of perception ability, our proposed algorithm has achieved better results, and the black artifacts caused by different imaging mechanisms have been greatly improved. To sum up, the result is consistent with the expectation and the algorithm is reasonable.

## 5. Conclusions and future work

In this paper, we proposed an IR and VI image fusion algorithm based on dual NSCT and PCNN. The direction information and filtered image features were weighted to stimulate PCNN, and the texture details in the VI image were fully extracted. It solves the problem that texture details are not fully fused in the fusion process. Compared with the optimal index of other three classical algorithms. In the first group experimental data, information entropy increased by 3%, standard deviation increased by 27%, average gradient increased by 20%, and spatial frequency increased by 16%. In the second group experimental data, information entropy increased by 2%, standard deviation increased by 5%, average gradient increased by 26%, and spatial frequency increased by 13%. In the third group experimental data, information entropy increased by 5%, standard deviation increased by 90%, average gradient increased by 41%, and spatial frequency increased by 27%. All above experimental data show that our proposed algorithm is a reasonable and effective IR and VI image fusion method.

At present, the development of convolutional neural network is in full swing, and it is widely used in the image field, such as the classification of CT images [30] and the prediction of harmful substances in the atmosphere [31]. Therefore, in the future work, we plan to apply CNN in image fusion. And through the trained CNN model to classify and extract the features of the image. As the same time, because there are many hyperparameters in CNN, we will utilize improved particle swarm optimization PSO [32] to optimize hyperparameters to simplify the model training process.
